# Long-Term Influence of Platelet-Rich Plasma (PRP) on Dental Implants after Maxillary Augmentation: Implant Survival and Success Rates

**DOI:** 10.3390/jcm9020391

**Published:** 2020-02-01

**Authors:** Sameh Attia, Clara Narberhaus, Heidrun Schaaf, Philipp Streckbein, Jörn Pons-Kühnemann, Christian Schmitt, Friedrich Wilhelm Neukam, Hans-Peter Howaldt, Sebastian Böttger

**Affiliations:** 1 Department of Cranio Maxillofacial Surgery, Justus-Liebig University Giessen, Klinik Str. 33, 35392 Giessen, Germany; Clara-Narberhaus@web.de (C.N.); schaaf@mkg-am-theater.de (H.S.); Philipp.Streckbein@uniklinikum-giessen.de (P.S.); hans-peter.howaldt@uk-gm.de (H.-P.H.); Sebastian.Boettger@uk-gm.de (S.B.); 2 Medical Statistics, Institute for Medical Informatics, Faculty of Medicine, Justus-Liebig University Giessen, Rudolf-Buchheim Str. 6, 35392 Giessen, Germany; Joern.Pons@informatik.med.uni-giessen.de; 3 Department of Oral and Maxillofacial Surgery, University of Erlangen, Glückstr. 11, 91054 Erlangen, Germany; schmitcn@outlook.de (C.S.); Friedrich.Neukam.extern@uk-erlangen.de (F.W.N.)

**Keywords:** implant survival, implant success, PRP, platelet-rich plasma, long-term result, sinus lift

## Abstract

The atrophic maxilla often requires bone augmentation before implant placement to ensure long-term implant success. A previous prospective clinical trial examined the use of platelet-rich plasma (PRP) during maxillary augmentation. The short-term results showed no positive effect of PRP. The aim of this study was to evaluate the same patient collective of the previous study regarding the PRP long-term impact on the survival and success of dental implants. Fifty-three patients from the previous study diagnosed with maxillary atrophy and augmented with autologous bone grafts from the iliac crest and dental implants, were included in this study. Treatment was carried out on both sides in 34 patients with a split-mouth-design in which one randomly chosen side was treated additionally with PRP, the other side was the control-side. Nineteen patients were treated only on one side and were assigned to the PRP—or the control group randomly. Implant follow-up of the patients from the previous study was performed after an average time of 13 years. Implant success was evaluated using two different success criteria. Thitry-seven patients (25 women and 12 men) were investigated in this study. Seventeen patients (12 female, 5 male) were included in the PRP group, while 20 patients (13 female, 7 male) participated in the control group. A total of 210 implants were inserted. Of these, 102 implants (48.57%) were placed in the PRP group and 108 implants (51.42%) in the control group. Out of 102 investigated implants in the PRP group, 6 were removed (survival rate 94.1%). While two of the 108 implants in the control group were loss (survival rate 98.1%). In the PRP group, the cumulative probability of survival after 15.1 years was 94.1% and in the control group, was 98.1%, with no significant difference between the two groups. Higher significant difference for the control group was found in the cumulative success probability using Albrektson criteria (*p* = 0.05). Positive impact of PRP on long-term implant survival and success could not be found.

## 1. Introduction

Facial bone loss due to trauma, disease, aging or congenital abnormalities can lead to deficiencies of maxillary bone. Depending on its extent, it remains a challenge for both health professionals and patients. Furthermore, bone loss has a high psychological impact on patients. Therefore, aesthetic and functional reconstruction have the same importance in the maxillofacial region [1]. Tissue engineering is increasingly being used to reach excellent aesthetic and functional results of facial reconstruction. In this context, also platelet-rich plasma (PRP) is used to enhance autologous bone grafts [2]. The last years showed a staggering development concerning the use of tissue engineering and biomaterials such as decellularized matrix, nanoparticles, stem-cell therapies, scaffolds, and even the engineering of a whole tooth. This has shifted our beliefs and expectations regarding the results of bone reconstruction [3]. But the development of such new strategies has not yet been completed and therefore, older approaches such as PRP and platelet-rich Fibrin (PRF) still require validation or disproof of their clinical efficacy through long-term follow-ups [3,4,5].

Successful clinical implantation can only be performed with sufficient bone supply [6]. Enough bone in vertical and horizontal direction is necessary for the primary implant stabilization and lead to successful osseointegration [7,8]. Implants should be at least 10 mm in length and 3 mm in diameter [8]. Extensive, individualized prosthetic and surgical planning before implantation is desired as implants should be placed where they are needed for prosthetic rehabilitation [9,10]. However, the ideal implant site may be located in atrophic jaw areas. In such cases, bone augmentation is necessary prior to implantation [6]. In order to achieve better results of bone augmentation surgery and to reduce the impairment of the patients, a constant attempt is made to improve augmentation techniques and efficiency [11]. 

Marx et al. reported for the first time in 1998 that the intraoperative use of platelet-rich plasma (PRP) leads to faster bone healing in autologous graft transplantation [2]. Since then, many other studies have been conducted to investigate the effect of PRP in this context [12,13]. PRP is an autologous concentrate of human platelets in a small amount of plasma [14]. It has a 3 to 5-fold higher platelet concentration than normal blood [15]. The growth factors contained in platelets play a major role in wound healing and have been used in implantology to improve bone regeneration [12]. 

To demonstrate these effects, many studies examined the influence of PRP on augmented autologous bone. Aimetti et al. involved four patients undergoing bilateral bone augmentation in the upper jaw. Using split-mouth design, PRP was used on one side of the maxilla. The authors were able to demonstrate higher bone regeneration at the PRP side [16]. Consolo et al. investigated the effect of PRP on bone regeneration in 17 patients. Patients received bilateral sinus lift surgery with autologous bone graft. This was enriched with PRP on one side. Radiological and histological evaluation of bone density was performed. The authors documented increased bone density and activity on the PRP side. However, they also observed a diminished positive effect of PRP approximately six months post intervention [17]. The same design of the previous study was used by Khairy et al. in 15 patients. After three months, the authors could not find a positive effect of PRP, but they were able to detect increased bone mineral density in the PRP group after six months [18]. The clinical question of Bettega et al. was whether the use of PRP in sinus lift surgeries with autologous bone leads to higher bone formation [19]. Bilateral treatment of 18 split-mouth patients was performed. The histological and radiographical bone examination did not show statistically significant differences between the two groups [19]. The research by Raghoebar et al. examined the effect of PRP on the remodeling of autologous bone grafts. Two-sided jaw bone augmentation was performed in five patients using PRP on a randomly selected jaw side. The authors were unable to document any improvement in wound healing or bone remodeling when using PRP [20]. Consequently, the presented studies could not clearly demonstrate the efficacy of PRP in augmentation surgery of jaws. According to Aurora et al., this is mainly due to the lack of standardization of the study structure and small population sizes [21]. 

In order to investigate the influence of PRP on bone density, a bi-centric clinical randomized study was conducted at the Departments for Craniomaxillofacial Surgery in Giessen and Erlangen, Germany. Between 2001 and 2004, 53 patients with maxillary atrophy were included in the study. Sinus lift operation was performed, if necessary, in combination with an on-lay graft osteoplasty. Patients were randomly assigned to the study group (sinus lift augmentation with PRP) or to the control group (augmentation without PRP). Nineteen patients underwent unilateral surgery and the remaining 34 were treated bilaterally (split-mouth design). PRP concentrations added to the cancellous bone used to fill the maxillary sinus were measured. The intraoperative procedure is as follows:

1. 3 mL PRP (drops) on 3 cm^3^ of cancellous bone;
2. 0.5 mL calcium gluconate 10% (drops) on 3 cm^3^ of cancellous bone;
3. 0.5mL of native wound blood (drops) on 3 cm^3^ of cancellous bone.

The substance was gently resuspended with a plastic pipette until, after about four minutes, a homogenous gel-like consistency was formed. This allowed better handling of the bone graft. After a healing period of four months, the implants were inserted using a drilling template. This was created by 3D planning with CT scans made during the healing period. During implant surgery, biopsies were taken from the graft region on each side treated. The biopsies were evaluated histomorphometrically for bone density. Bone density was also examined on CT by means of the Hounsfield scale in the augmented regions. The results showed no significant differences between the study groups. Neither the histomorphologically nor the radiographically assessed bone density was significantly higher with the use of PRP. Similarly, the rate of short-term implant loss, graft resorption, and clinical parameters did not vary significantly between the groups. Implant loss was evenly distributed in both groups. Schaaf et al. concluded that PRP has no short-term effect on bone density and bone regeneration [22,23].

Long-term effects of the use of PRP in combination with autologous bone augmentation has on implantation success is not well reviewed in literature. To date, few studies can be found which only marginally address this topic [24,25,26,27,28]. Between 2004 and 2017, many studies dealing with the long-term impact of PRP on implant survival and success were published. Velich et al., 2004, examined the long-term results of various augmentation materials during sinus lift operations. In one out of ten investigated groups, bone augmentation was carried out with alloplastic bone substitute material and PRP. The follow-up period for this study was up to five years. An implant loss rate of 3.6% was recorded in the PRP group but this was not statistically significant in comparison to the other groups. Robiony et al., 2008, investigated the use of PRP after distraction osteogenesis, autologous bone transplantation and insertion of dental implants. Twelve patients who received such treatment were examined; there was no control group. At a follow-up time of five years post dental implants, a survival rate of 97.9% and a success rate of 91.5% were recorded according to Albrektsson. In a split mouth design examined by Dasmah et al. in 2013, dental implants which were inserted after iliac bone graft with and without PRP were examined. After an observation period of five years, there was no implant loss and therefore no statically significant data between the groups could be found. Long-term implant survival after maxillary bone augmentation using intraorally obtained autologous bone and xenogeneic bone substitute mixed with PRP was presented by Schwartz-Arad et al. in 2014. The study was conducted without a control group and stated an implant survival rate of 93.4% after an average observation time of 3.3 years. Finally, in 2017, the study published by Khouly et al. stated a cumulative survival of dental implants inserted after sinus lift augmentation with xenogeneic bone and PRP of 89.9% at an average of 7.2 years post-surgery. The study did not contain a control group.

The existing literature findings do not allow a valid statement on the long-term results of implant survival and implant success after the use of PRP. The aim of this study was to determine whether the use of PRP during augmentation of the maxilla has a long-term impact on implant treatment outcome. Implant survival and success rates were assessed. From the multitude of success criteria available in the literature, the criteria according to Albrektsson et al. [29] and Buser et al. [30] were considered to be most useful. They represent the most frequently used criteria for assessing implantation success [31]. This allows a comparison of implant success results with other studies using the same criteria. To date, an official uniform and international agreement on how to define success in implantology and related criteria is still lacking [32]. In this study, we hypothesized that implants placed in PRP-enriched augmented bones lead to higher survival and success rates.

## 2. Materials and Methods

### 2.1. Study Design and Patient Population

The study was conducted as a two-center, controlled randomized single-blind retrospective study which builds on the research of Schaaf et al. [22,23]. The inclusion criterion of this study was the participation in the previous randomized controlled trial (RCT). However, patients with pregnancy and reduced general medical and physical condition were excluded. All investigated implants were assigned to either the study (inserted in PRP treated bone) or control (without PRP) group. Due to the identicality of patients’ factors, implants inserted bilaterally in a split mouth design were evaluated separately. 

### 2.2. Examiner Blinding and Calibration

The clinical examinations and radiological evaluations were carried out by one examiner who did not know the patients’ research group assignment, so blinding was ensured. In order to ensure the validity of measurements, the examiner was trained prior to data collection. For randomly selected patients, all clinical and radiological parameters to be recorded in the study were measured on a total of 50 implants and compared with the results of an experienced oral surgeon. 

### 2.3. Study Parameters

The main parameter was the long-term survival and success rate of the implants. Implants that remained in situ were defined as surviving implants. To calculate the survival rate, the existing implants were noted during the clinical examination and the number and time of removed implants were documented. Since implant survival is not equivalent to implant success [33,34], success criteria of Buser et al. [30] and Albrektsson et al. [29] were evaluated in this study. Albrektsson et al. defined dental implant success as a state of no clinical implant mobility or radiographic radiolucency, annual vertical bone loss of less than 0.2 mm after the first year post surgery, and absence of irreversible symptoms such as pain, infection, neuropathy, paranesthesia or mandibular canal injury [29]. Buser et al. include the following aspects to rate an implant as a success: no subjective complaints (pain, foreign body sensation or dysesthesia), no recurrent purulent infection, no mobility, no continuous radiolucency around the implant, and possibility of prosthetic loading [30]. The main difference between the two known criteria is considering the vertical bone loss as a success factor. In order to assess implant success, the implants were examined with regard to the corresponding criteria. If one or more of the negative criteria were present, the implant was considered a failure. Already explanted implants were also rated as a failure.

### 2.4. Statistical Analysis

Collected data was divided into two groups (bilaterally and unilaterally treated patients) prior to statistical analysis. In each group, results of the PRP-group were compared with the results of the control group. Group similarity was compared by using the rank sum test according to Mann–Whitney and Wilcoxon. In order to compare frequencies, Fisher’s exact test or the chi-square test was used, depending on the available data. Survival and success rates were analyzed with the Kaplan–Meier method. Implants that were in situ or successful during the observation period are censored and thus included in the calculation of survival and success probabilities [35]. The survival and success probabilities were compared with the logrank test. For all statistical tests applied, the significance level was defined as α = 0.05. Therefore, for a *p*-Value above 0.05, the null hypothesis was retained, for values below 0.05, the alternative hypothesis was adopted.

### 2.5. Ethics and Privacy

The study protocol was presented and approved by the ethics committee of Justus-Liebig University Giessen (approval number 129/15). The patients consented that their intraoral pictures and X-ray images may be used anonymously in the publications. In addition, all data in the Excel spreadsheet was pseudonymized. 

## 3. Results

Out of 53 participants, 37 patients in two centers (Giessen 31 out of 41, Erlangen six out of 12) were available for follow-up examination and included in this study. Reasons for the drop-out of 16 patients were: rejection of participation (*n* = 7), failure to reach patients (*n* = 5), poor medical condition (*n* = 2), and decease (*n* = 2). Out of the 37 examined patients, 25 (67.6%) were female and 12 (32.4%) male. Patient age ranged between 30 and 90 years, the median was 65 years. In the study by Schaaf et al., 306 implants were originally placed. Due to the withdrawal of 16 patients, 96 implants could not be examined. Consequently, 210 implants were considered in the statistical evaluation, which represents 68.6% of the original implant number. Due to eight implant losses, 202 implants were clinically and radiographically examined. Reasons for implant loss (*n* = 8) were documented as follows: periimplantitis (*n* = 5, 4 PRP side,1 control side), removal of implant at the PRP side due to intra-operative complication (perforation of the maxillary sinus, *n* = 1), and failure of osseointegration (*n* = 2). The implant observation period was between 11.3 and 15.1 years. The median and mean age of implants was 13 years. Regardless of control or PRP group, a survival rate of 96.2 percent could be calculated. 

**Figure 1:**
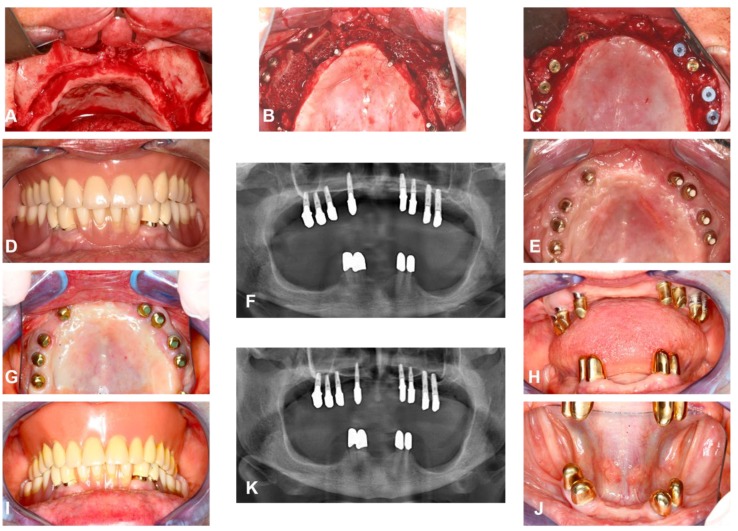
Clinical case included in the study. (**A**,**B**) intra-operative images show extrem maxillary atrophy before and after autologus bone transplantation from the iliac crest and bilaterial sinus lift, on one side platelet-rich plasma (PRP) was used, (**C**) intra-operative image, six months post implant placement (*n* = 8), (**D**–**F**) intra-oral image and panoramic radiograph after prosthetic rehabilitation, (**G**–**J**) intra-oral images and panoramic X-ray at long-term follow-up examination at 13 years post-surgery.

A case presentation of one of the included patients can be seen in Figure 1.

### 3.1. General Implants Evaluation

The general implants evaluation includes all inserted implants in 37 patients: 23 bilaterally treated patients and 14 patients treated on one side. Implants were assigned either to PRP or control group. 

#### 3.1.1. Patients Gender, Age, and Smoking Behavior 

Seventeen patients (12 female, 5 male) were included in the PRP group, while 20 patients (13 female, 7 male) participated in the control group. Uniform gender distribution between the groups was demonstrated with Fisher’s exact test (*p* = 1.0). The age similarity of the patients in the PRP (mean 65.9, SD 16.6, median 68) and control group (mean 60.1, SD 15.8, median 63.5) was given (*p* = 0.25). Patient’s information about smoking behavior was evenly distributed as Fisher’s exact test indicated *p* = 0.67 for the structural similarity of the two groups.

#### 3.1.2. Implant Numbers and Survival Age 

A total of 210 implants were inserted. Of these, 102 implants (48.57%) were placed in the PRP group (mean 12.5, SD 2.7, median 13.0 years) and 108 implants (51.42%) in the control group (mean 12.9, SD 1.6, median 13.0 years). Fisher’s exact test demonstrated homogeneity of the two groups with respect to the number of implants per group (*p* = 0.16). The structural similarity between the groups was proven by Fisher’s exact test (*p* = 0.16). The rank sum test according to Mann–Whitney and Wilcoxon with *p* = 0.76 proved the structural similarity between the PRP and the control group.

#### 3.1.3. Survival Rate of Implants

**Figure 2:**
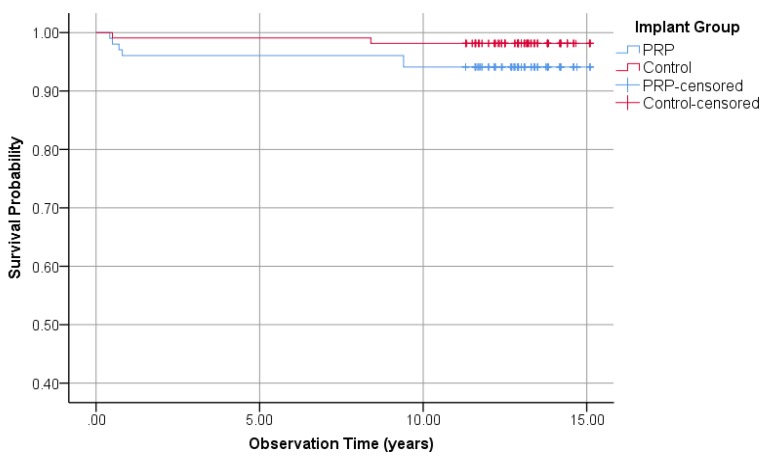
Kaplan–Meier survival probabilities of 210 investigated implants, 102 PRP, and 108 control group.

Out of 102 investigated implants in the PRP group, 6 were removed (survival rate 94.1%). While two of 108 implants in the control group were lost (survival rate 98.1%). According to Fisher’s exact test with *p* = 0.16, no difference between the two groups could be found. The Kaplan–Meier curve in Figure 2 shows the survival time analysis for all implants assigned to the PRP and control group. In the PRP group, the cumulative probability of survival after 15.1 years was 94.1%. In the control group, the cumulative probability of survival was 98.1%. The comparison of survival times using the logrank test resulted in *p* = 0.08 and therefore, no significant difference between the two groups was observed.

#### 3.1.4. Implant Success Rate Regarding Buser 

**Figure 3:**
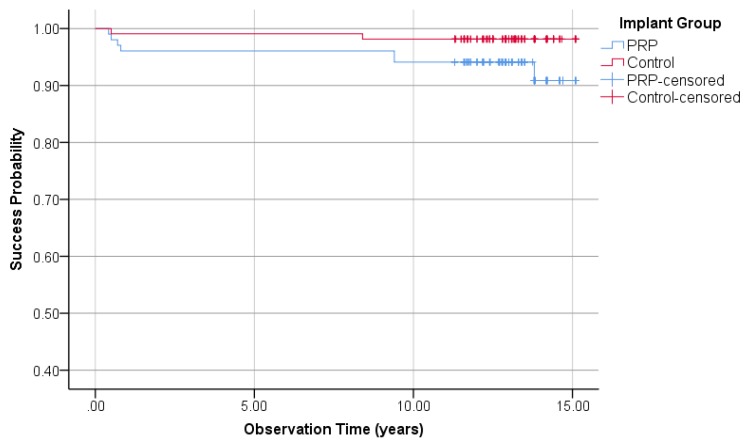
Kaplan–Meier success probabilities of 210 implants according to Buser implant success criteria.

Regarding the Buser implant success criteria, 7 implants in the PRP group and two implants in the control group failed to fulfill the success requirements and were rated as failures. This led to a success rate of the PRP and control group of 93.1% and 98.1%, respectively (Fisher’s exact test: *p* = 0.94). Kaplan–Meier cumulative success probabilities of the PRP group after an observation period of 15.1 years is 90.9%. The last variable in the control group after 15.1 years was 98.6%. The logrank test was used to compare the success probabilities. A difference between the PRP and the control group could not be determined (*p* = 0.13, Figure 3).

#### 3.1.5. Implant Success Regarding Albrektsson

**Figure 4:**
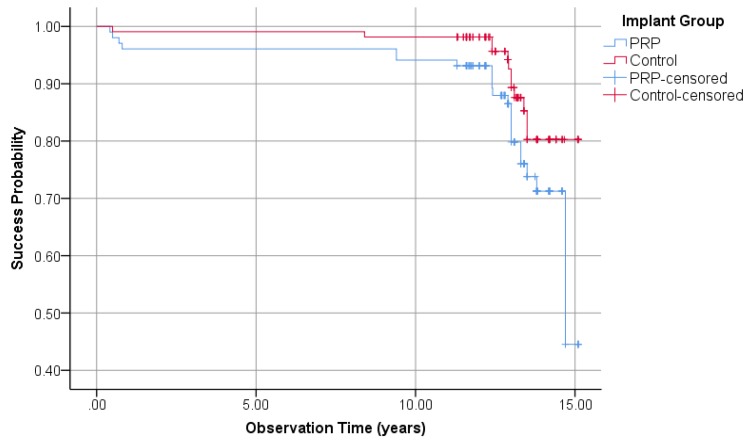
Kaplan–Meier success probabilities of 210 implants according to Albrektsson implant success criteria.

By applying the Albrektsson implant success criteria on the PRP and control groups, 23 and 12 implants were unsuccessful, respectively. This results in success rates of 77.5% in the PRP group and 88.9% in the control group (Fisher’s exact test: *p* = 0.04). Kaplan–Meier cumulative success probability in the PRP group after 15.1 years was 44.5%, whereas the cumulative success probability in the control group was 80.3% (Figure 4). The logrank test showed a significant difference at borderline level between the PRP and control groups (*p* = 0.05). 

### 3.2. Split-Mouth Evaluation

The split-mouth evaluation included only the 23 patients who were treated on both sides of the maxilla. One hundred and seventy-one implants were inserted (90 PRP side and 81 control side). Fisher’s exact test represents a uniform distribution (*p* = 1.0). 

#### 3.2.1. Survival Rate of the Implants (Split-Mouth)

**Figure 5:**
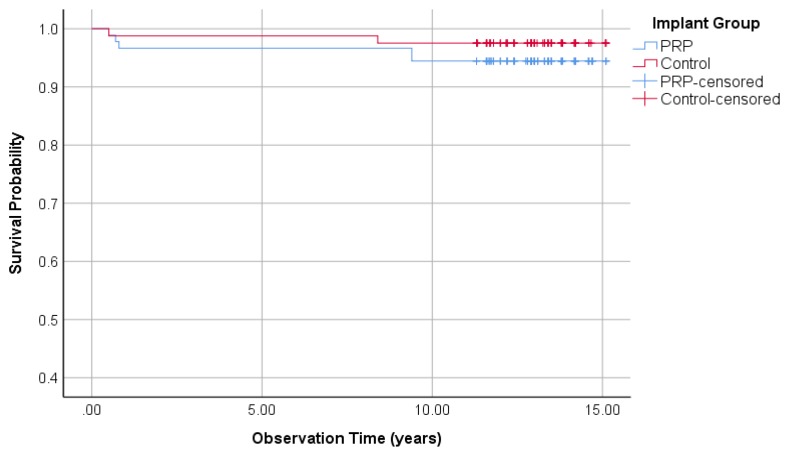
Survival rate of 171 implants in PRP and control side (split-mouth).

Altogether, seven implants were lost on the PRP-side, 5 out of 90 implants were removed (survival rate 94.4%). On the control-side, two out of 81 implants were lost (97.5% survival rate). The cumulative survival probability according to Kaplan–Meier after an observation period of 15.1 years in the PRP and control side was 94.4% and 97.5%, respectively. The logrank test with *p* = 0.31 showed no statistically significance between the PRP and the control side regarding survival rates of the implants (Figure 5).

#### 3.2.2. Implant Success Rate Regarding Buser (Split-Mouth)

**Figure 6:**
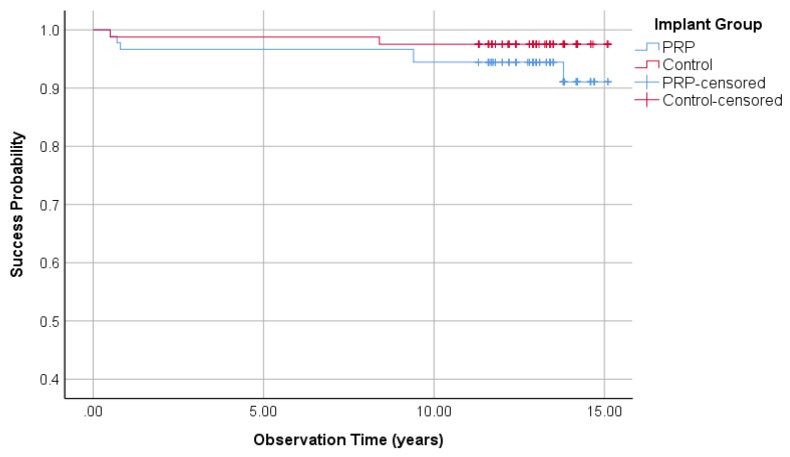
Kaplan–Meier success probabilities of 171 implants regarding the Buser implant success criteria in the split-mouth evaluation.

On the PRP side, six implants failed to meet the success criteria of Buser and were rated as failure, resulting in a success rate of 93.3%. The control side had two unsuccessful implants (97.5 % success rate). Kaplan–Meier success probability after an observation period of 15.1 years in the PRP and control side was 91.1% and 97.5%, respectively. Despite better results on the control side, there was no statistically significant difference between the two sides regarding Buser implants success tested by the logrank test (*p* = 0.20) (Figure 6).

#### 3.2.3. Implant Success Regarding Albrektsson (Split-Mouth)

**Figure 7:**
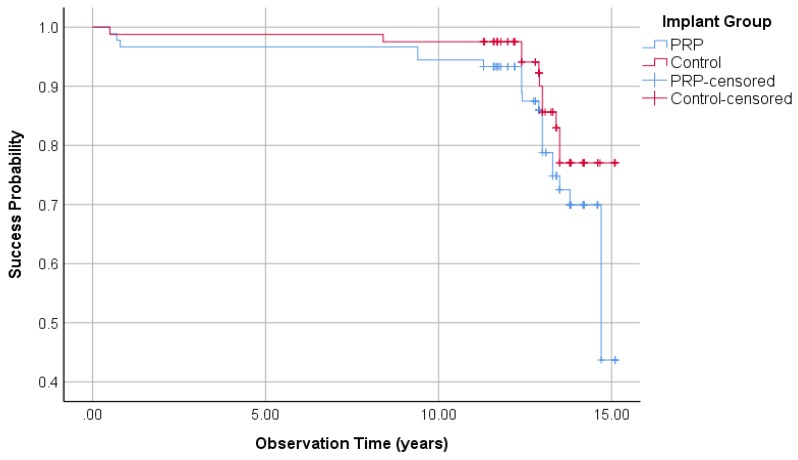
Kaplan–Meier success probabilities of 171 implants regarding the Albrektsson implant success criteria in the split-mouth evaluation.

On the PRP side, 22 implants were unsuccessful versus eleven implants on the control side referring to the Albrektsson implant success criteria. Consequently, the success rates of implants at the PRP and control side are 75.6% and 86.4%, respectively. Figure 7 visualizes the Kaplan–Meier success probabilities regarding to the Albrektsson criteria. On the PRP side, the 5-, 10-, and 15-year success probability was 96.7%, 94.4%, and 43.7%, respectively. The results on the control side were 98.8%, 97.5%, and 77%. Within the logrank test, a *p*-Value of *p* = 0.13 showed no significant difference of success rate between the PRP and the control side.

### 3.3. Overall Evaluation

Table 1 presents the 12 examined parameters of the general and split-mouth evaluation. Overall, the values from the PRP group were worse than the control group. 

## 4. Discussion

The aim of this study was to examine whether PRP has a positive influence on the long-term survival and success of dental implants when used in combination with maxillary bone augmentation. This could not be confirmed by the evaluation of the three parameters (survival rate, success rate according to Buser criteria, and Albrektsson criteria). Most parameters did not show any significant difference between the PRP group and the control group. Kaplan–Meier cumulative success probability according to Albrektsson criteria was statistically significantly higher in the control group than the PRP group. Thus, the hypothesis could not be proven.

Results presented in this study follow the findings of the previous study by Schaaf et al. and prove (in the same patient collection) that PRP has no positive effect in the use of maxillary augmentation on short-term and long-term results. The goal of bone augmentation is to create an optimal bone situation for implant placement and osseointegration, thus ensuring long-term implant success. This study did not show any positive influence of PRP on the long-term implant results. However, it should be noted that the implant success rates of both groups can be regarded high, and thus very satisfactory after an average of 13 years. To see any beneficial effect for any treatment modality, a high number of patients are needed which is a challenge to achieve. It is therefore without surprise that no significant difference in treatment outcome was found.

The most evaluated variable in literature concerning the long-term implant outcome after jaw augmentation and PRP treatment is implant survival. Only one study (Robiony et al.) evaluated the implant success according to Albrektsson criteria and reported an implant success rate of 91.5% after five years. Bone augmentation prior to implantation was performed in combination PRP [27]. In this study, the results regarding long-term survival of the PRP group are inferior. Two reasons might cause this effect. First, the longer follow-up period of this study has to be considered. Second, in the study by Robiony et al., no control group was included. This represents a significant disadvantage because only with a controlled and randomized study design the benefit of a treatment modality, here PRP, can be validly proved as effective [36].

The overall analysis (Table 1) shows that the values of all parameters in the PRP group is inferior to the control group. Therefore, the question arises whether PRP may have a negative impact on long-term outcome. The failure rate probabilities according to Albrektsson criteria were distinctly higher in the PRP group. In order to clarify whether PRP had a negative influence on long-term implant outcome, further controlled trials with larger numbers of test subjects might prove this hypothesis.

In the present study, the two internationally recognized success criteria of Buser and Albrektsson were used. The success rates differed depending on which success criterion was applied. Generally, Albrektsson success criteria presented lower success rates as the one of Buser. The reason may be the assessment of the peri-implant bone loss. However, both success criteria have deficits because they do not reflect the condition of the implant and the surrounding tissue sufficiently. Furthermore, patient satisfaction is not addressed in either success criteria. Some papers suggested the development of a success score [34,37] to assess clinical, radiological, prosthetic and patient satisfaction parameters, as well as implant survival. In addition, an internationally recognized score to measure implant success is highly beneficial to compare the success rates in different studies. 

Recently published meta-analysis concerning long-term implant survival after sinus lift surgery and augmentation using different bone grafts conclude that there is no significant difference between autologous and substitute bone graft [38,39]. The current study results also confirm this finding. Therefore, further clinical trials dealing with other regenerative therapy strategies should be conducted. Many of these strategies were described in recent literature [3,4,40]. Most of them aim to avoid surgical complications such as donor-site morbidity in case of autologous bone transplantation and limitations of PRP-technique. Practical considerations regarding feasibility, ethics, and specialization are also relevant when planning a new therapeutic regimen.

The recent discovery of generating mesenchymal stem cells from periapical tissue is a promising concept and might be available for dental practitioners in the future [41]. Due to the good porosity at low costs, scaffolds are often used in cases of disturbed bone healing and in setting of tumorous bone disease [40]. Furthermore, the use of mineral agents like calcium phosphate cement added with active ingredients such as strontium or nanoparticles loaded on scaffolds such as nanosilicates (poly (glycerol sebacate)) showed an improved mineralization in vivo and in vitro [4,42]. Beside the long way until becoming a certified product, all the above-mentioned technologies are still in the early stages of development with many unknown factors. Long-term follow-up studies are required to demonstrate their clinical effectiveness. Probably it will be time-consuming and hard to prove the benefit of these treatment modalities in RCT. Thus, even older techniques like PRP should be further developed and revalidated. This long-term follow-up study aims to assess the value of autologous bone augmentation in combination with and without PRP, which has been a state-of-the-art approach for many decades.

## 5. Conclusions

Long-term survival and success rates of dental implants placed after sinus-lift surgery using autologous bone is high and similar to various other bone substitute materials. PRP shows no positive impact with a tendency to a slightly negative effect on implant survival and other success criteria. Therefore, upcoming biologic regenerative therapies to improve bone healing and implant-tolerance should be investigated further.

## Figures and Tables

**Table 1:** Summary of test results and comparison of PRP and control group based on tested parameters.

Parameter		PRP-Group		Control-Group	
		Value	Rating	Value	Rating
**General-Evaluation**	Survival rate	94.1%	-	98.1%	+
	Cumulative survival rate	94.1%	-	98.1%	+
	Success according to Buser	93.1%	-	98.1%	+
	Cumulative success according to Buser	90.9%	-	98.6%	+
	Success according to Albrektsson	77.5%	-	88.9%	+
	Cumulative success according to Albrektsson	44.5%	-	80.3%	+
	Mean years of Evaluation	12.53		12.90	
**Split-Mouth-Evaluation**	Survival rate	94.4%	-	97.5%	+
	Cumulative survival rate	94.4%	-	97.5%	+
	Success according to Buser	93.3%	-	97.5%	+
	Cumulative success according to Buser	91.1%	-	97.5%	+
	Success according to Albrektsson	75.6%	-	86.4%	+
	Cumulative success according to Albrektsson	43.7%	-	77%	+
	Mean years of Evaluation	13.1		13.1	

+: Value is better than in the other group, -: Value is worse than in the other group.
